# Radioprotective effect of omeprazole against testicular damage induced by ionizing radiation in mice: An experimental study

**DOI:** 10.18502/ijrm.v23i1.18202

**Published:** 2025-03-21

**Authors:** Ali Nabi, Azam Hassanpour Dehnavi, Fahime Mazaheri, Nastaran Momeni, Habib Nikukar, Mahdie Hemati, Seyed Jalal Hosseinimehr, Masoud Shabani, Fatemeh Sadeghian-Nodoushan, Javad Biabani-Ardakani

**Affiliations:** ^1^Research and Clinical Center for Infertility, Yazd Reproductive Sciences Institute, Shahid Sadoughi University of Medical Sciences, Yazd, Iran.; ^2^Andrology Research Center, Yazd Reproductive Sciences Institute, Shahid Sadoughi University of Medical Sciences, Yazd, Iran.; ^3^Department of Anatomical Sciences and Cell Biology, Medical Faculty, Shahid Sadoughi University of Medical Sciences, Yazd, Iran.; ^4^Biotechnology Research Center, Yazd Reproductive Sciences Institute, Shahid Sadoughi University of Medical Sciences, Yazd, Iran.; ^5^Shahid Ramezanzadeh Radiation Therapy Center, Shahid Sadoughi University of Medical Sciences, Yazd, Iran.; ^6^School of Advanced Medical Sciences and Technologies, Shahid Sadoughi University of Medical Sciences, Yazd, Iran.; ^7^Department of Clinical Biochemistry, Faculty of Medicine, Shahid Sadoughi University of Medical Sciences, Yazd, Iran.; ^8^Department of Radiopharmacy, Faculty of Pharmacy, Mazandaran University of Medical Sciences, Sari, Iran.; ^9^Department of Radiation Oncology, School of Medicine, Shahid Sadoughi University of Medical Sciences, Yazd, Iran.

**Keywords:** Omeprazole, Ionizing radiation, Radioprotective, Radiotherapy, Testicular.

## Abstract

**Background:**

Radiation-induced normal tissue damage remains a major concern in radiotherapy, particularly affecting rapidly dividing cells, including those in reproductive tissues. Developing effective radioprotective agents to mitigate this damage is crucial for preserving fertility.

**Objective:**

The radioprotective effects of omeprazole (OMP) were investigated in adult male mice undergoing external radiation.

**Materials and Methods:**

In this experimental study, 36 adult male mice (30–35 gr, 6–8 wk old) were divided into 6 groups and orally administered OMP daily via oral gavage for 7 days before whole-body irradiation. On the 8^th^ day, mice were subjected to a single 6 Gray dose of 6 megavoltage X-ray radiation. Blood samples were collected via cardiac puncture for testosterone level evaluation, while testicular specimens were harvested post-euthanasia for sperm parameters assessment and histological analysis. Additionally, spermatogenic cell density was evaluated.

**Results:**

Irradiation of 6 Gray X-ray to the testis of mice significantly affected sperm count, progressive motility, DNA fragmentation, the number of sperm with normal morphology, and the number of immotile sperm. Furthermore, administration of OMP improved progressive motility, DNA fragmentation, and sperm viability. Histopathological findings showed irradiation led to severe testicular atrophy with spermatogenic arrest and abnormal cytoarchitecture vacuolation and interstitial edema, while OMP treatment reversed relative radiation toxicity, especially in the 50 mg OMP treatment group.

**Conclusion:**

In conclusion, OMP could act as an effective radioprotector against testicular damage following X-ray irradiation in an animal model. Further studies are needed to investigate OMP potential in protecting human testis tissue.

## 1. Introduction

Radiotherapy has consistently proven to be a highly effective method for treating cancer, with approximately 50% of cancer patients undergoing radiotherapy at various stages of their therapeutic management, both for curative and palliative aims (1). The radiation used in radiation therapy is recognized as ionizing radiation, as it can produce electrically charged ions and release energy within cellular structures as it passes through. The fundamental objective of radiation therapy is to maximize the radiation dose to abnormal cancer cells while minimizing exposure to normal cells, which are adjacent to cancer cells or in the path of radiation.

However, through the delivery of high radiation doses to the tumor sites, there are concerns about damage to the surrounding healthy vital organs (2), and they can cause side effects with varying severity and duration (3). Among various body tissues, the testis is one of the most radiosensitive ones. In humans, doses as low as 0.1 Gray (Gy) leads to a temporary reduction in the number of spermatogonia, and a dose of 0.15 Gy will result in temporary infertility. After a radiation dose of 2 Gy, azoospermia occurs for several years, and permanent azoospermia is the result of a dose in the range of 6–8 Gy with 2 Gy per session (4). Recovery is very slow in men. It takes a long time for sperm production to recover, over 6 months after a dose of 1.0 Gy and possibly more than 2 yr after 6.0 Gy. Even after sperm count recovers, fertility issues or miscarriages can happen due to low-quality or genetic anomalies (5).

Efforts are being made to reduce the side effects of radiation therapy. One approach is improving the precision of radiation delivery with techniques like intensity-modulated radiotherapy and proton therapy (6). These methods help but cannot completely protect healthy tissues near the tumor. Another approach involves using compounds, either synthetic or naturally occurring, to help healthy tissues better respond to radiation (7).

There are 4 main groups of such agents: synthetic (8), immunomodulator (9, 10), natural (11, 12), and herbal (13). These agents have various abilities, like reducing the damage of free radicals, acting as antioxidants, stimulating the immune system, and toxicity reduction properties (8). Several recent studies have focused on repurposing current medicine as radioprotective agents. A good choice should protect most organs, be easy to use, have minimal side effects, be stable for a long time, and be compatible with other medications the patient might be taking. Among various candidates, omeprazole (OMP), a proton pump inhibitor (PPI) frequently used to treat gastrointestinal disorders, looks promising because of its antioxidant and anti-inflammatory properties. Studies have shown that PPIs have a protective effect on normal tissues in the irradiated area by reducing the inflammatory response (14). Moreover, in vivo studies demonstrate the use of antioxidants as effective radiation protectors (15–17). In addition to the above properties, OMP's low risk of toxicity and widespread availability make it a good option for radiation protection.

While these findings look promising, there remains a need for a comprehensive understanding of its mechanisms of action, optimal dosage, and timing of administration. To address these questions, this study aims to evaluate OMP's radioprotective properties against testicular damage induced by ionizing radiation in mice. The result of this study holds the promise of providing valuable insights into whether OMP could serve as a viable treatment for radiation-induced testicular damage in humans, as well as the optimal protocols for its administration.

## 2. Materials and Methods

### Animals

This experimental study was performed in Yazd Reproductive Sciences Institute, Yazd, Iran from January to December 2023. A total of 36 adult male mice (30–35 gr body weight, 6–8 wk old) were obtained from the Animal Research Center of Biotechnology, Yazd Reproductive Sciences Institute, Yazd, Iran, and were acclimatized for a week before the study. Animals were kept based on guidelines for the care and use of laboratory animals in Iran in standard conditions of 12-hr light/dark cycle with relative humidity (50 ± 10%) and temperature (26 ± 2 C). Mice received standard food and water and were kept in polypropylene cages.

### Study design

Animals were randomly divided into 6 groups (n = 6/each):

G1 (Control): Mice received a 0.5% solution of carboxymethyl cellulose containing 0.9% NaCl.

G2 (Radiation): Mice were only exposed to total body radiation with a dose of 6 Gy.

G3 (OMP 30 mg/kg): Mice received 30 mg/kg daily for 7 days.

G4 (Radiation + OMP 30 mg/kg): Mice were pre-treated with OMP at a dose of 30 mg/kg daily for 7 days, and on day 8, they were exposed to 6 Gy whole-body irradiation.

G5 (OMP 50 mg/kg): Mice received 50 mg/kg daily for 7 days.

G6 (Radiation + OMP 50 mg/kg): Mice were pre-treated with OMP at a dose of 50 mg/kg daily for 7 days, and on day 8, they were exposed to 6 Gy whole-body irradiation.

**Figure 1:**
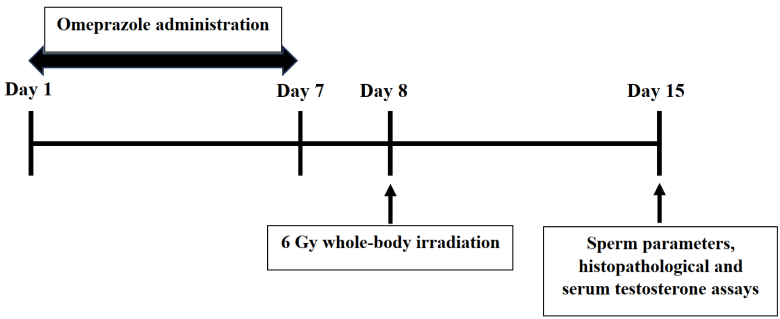
Study design diagram for evaluating the effect of OMP on testicular-induced irradiation. OMP was prescribed for 7 consecutive days, mice were exposed to 6 Gy whole-body irradiation on day 8, sperm parameters, histopathological, and serum testosterone assays were performed on day 15 of the study.

Animals in all groups were killed on day 15 of the study (1 wk after irradiation) and sperm parameters, histopathological, and serum testosterone assays were performed (Figure 1).

### Sample size 

The sample size was determined based on previous studies (18, 19).

### Drug preparation and irradiation

OMP (Tehran Chemistry Pharmaceutical Co., Iran) was freshly prepared in a 0.5% solution of carboxymethyl cellulose containing 0.9% NaCl and administered daily with 2 doses by oral gavage a week prior to whole-body irradiation. On day 8 of the study, mice were placed in a plexiglass phantom. They were irradiated with a 6 megavoltage X-ray beam produced by a radiotherapy machine (Linear accelerator, Siemens, Oncor, Germany) at a distance (source to skin distance) of 100 cm at a single dose of 6 Gy whole-body studies (20).

### Specimen collections

Mice from each group were fully anesthetized with an intraperitoneal injection of a mixture of ketamine (50 mg/kg, Alfasan, Netherlands) and xylazine (5 mg/kg, Alfasan, Netherlands), and blood samples were obtained from their hearts using a syringe to measure testosterone, and they were allowed to clot. The serum was centrifuged at 3000 rpm for 15 min and stored at -20 C for evaluating the testosterone. To collect testicular samples, immediately after euthanizing, the abdominal wall was opened, and then both testicles and epididymis were removed from the scrotum, and their weights were recorded. Testicles were placed in a 10% formalin fixative for histological section preparation.

### Sperm parameters evaluation

#### Sperm preparation and treatment

For sperm collection, the left and right epididymis were dissected and washed with saline; then, the caudal part of it was used for the assessment of sperm parameters. For sperm preparation, the Hams F10 medium supplemented with albumin was pre-warmed at 37 C, CO_2_ 6% in the incubator. The epididymis caudal part was minced and incubated in media (5 ml) for 15 min.

#### Measurement of sperm count 

10 µl of sperm sample was placed on the Makler chamber slide, and sperm number in vertical and horizontal squares was counted. Then, the mean of 2 was recorded and expressed as 10^6^/ml.

#### Measurement of sperm motility

For sperm motility assessment, 10 µl of sperm sample was placed on a pre-warmed slide, and 100 sperm were evaluated at different microscopic fields by phase contrast microscope (Olympus, Japan) with × 400 magnifications. Progressive motile spermatozoa, non-progressive motile spermatozoa and immotile spermatozoa percentage were reported based on the World Health Organization 2021 instructions.

#### Measurement of sperm viability

Sperm viability was measured by eosin-nigrosine staining. The sperm sample (10 µl) was mixed with the same volume of eosin-nigrosine dye. The smear of each sample was prepared on slides, and after drying in air, the sperm viability was evaluated by phase contrast microscope (Olympus, Japan) under × 1000 magnification. Sperm with no color was counted as viable, and pink or red ones were considered dead sperm.

#### Measurement of sperm morphology

Diff-quik staining kit (Ideh Varzan Farad Co., Iran) was provided to assess sperm morphology. A smear was prepared, and a staining procedure was performed based on kit instructions. The morphology of 200 sperm were assessed at a magnification of × 1000 by light microscopy (World Health Organization, 2021). The percentage of normal spermatozoa was recorded.

### Measurement of sperm deoxyribonucleic acid (DNA) integrity

A Halo sperm kit (SDFA kit, Ideh Varzan Farad, Tehran, Iran) was provided to measure sperm DNA fragmentation. Briefly, a sperm sample (50 µl) was added to low-melting agarose. Then, 20 µl of the mixture was placed on a precoated slide and spread by using a coverslip. After that slides were kept at 4 C for 5 min. Coverslips were removed, and staining procedures were carried out based on kit protocol instructions. Then, light microscopy with a magnification of × 1000 was applied to evaluate the DNA statue of 200 sperm. Halo size was the indicator of DNA fragmentation rate. Large or medium size of halo showed no DNA fragmentation, and sperm with small or no halo showed fragmented DNA.

### Serum testosterone level

The serum testosterone level was assessed using a commercial enzyme-linked immunosorbent assay kit (ZellBio, GmbH, Germany, Cat No. ZB-10260C-M9648) following the manufacturer's instructions. Optical density at a wavelength of 450 nm with a reference wavelength of 620–630 nm was measured using an enzyme-linked immunosorbent assay reader (21).

### Histopathological analysis (light microscopy studies) 

At the end of the experiment, the testis specimens were removed and then fixed in a 10% buffered formalin solution for 3 days, respectively, at room temperature. After fixation, tissue samples were dehydrated in ascending grades of alcohol (ethanol) and then cleared in xylene, which was then embedded in paraffin wax. The tissues were processed for paraffin blocking out, and then sections were cut with a rotary microtone (RM 2525, Leica Biosystems, Germany). Afterward, 5 μm thick sections, mounted on glass slides, were deparaffinized and rehydrated through a descending grade of alcohol and water to be stained with hematoxylin and eosin (H&E) (Merck GmbH, Darmstadt, Germany) staining method.

For histopathological studies, the stained slides were evaluated in random order by a histologist, who was blinded to the experimental groups to evaluate spermatogenesis and histopathological changes. The testicular tissue was examined under a light microscope (Olympus, Tokyo, Japan), with the magnification of × 40, × 100, and × 400 and photographed using a Zeiss Microscopy system.

### Ethical Considerations 

All the experimental procedures used in the present study were approved by the Animal Ethics Committee of the Shahid Sadoughi University of Medical Sciences, Yazd, Iran (Code: IR.SSU.AEC.1401.015). The animal experimental protocols were supported by the guidelines for care and use of laboratory animals in Iran.

### Statistical Analysis

Statistical analysis for the obtained data was performed using version 19 Statistical Package for the Social Sciences (SPSS) (Chicago, IL). All the data normally distributed are presented as the mean ± standard deviation (SD). Analysis of variance (ANOVA), one-way ANOVA, and Tukey's post-tests were used to eliminate any outliers from the normal data distribution. Statistically significant differences were accepted as p < 0.05.

## 3. Results

### Effects of OMP on sperm parameters (count, motility, viability, and morphology)

Radiation significantly decreased sperm count compared to the control group (p < 0.05). Administration of OMP did not remarkably change sperm count. Radiation caused significant decreases in the percentage of progressive motile spermatozoa. OMP 30 mg/kg (OMP 30), OMP 50 mg/kg (OMP 50), Radiation+OMP 30 mg/kg (R+OMP 30) and R+OMP 50 mg/kg (R+OMP 50) groups resulted in significant increases in the percentage of progressive motile spermatozoa compared to the radiation group (p < 0.01). Moreover, the progressive motile spermatozoa percentage noticeably increased in the control group compared to the radiation group (p < 0.001). The percentage of nonprogressive motile spermatozoa did not significantly alter among different groups. Radiation significantly decreased sperm morphology compared to the control group (p < 0.05). Radiation resulted in significant decreases in the percentage of viable spermatozoa compared to the control group (p < 0.001). A significant increase was observed in viable spermatozoa percentage in the control, OMP 30, OMP 50, and R+OMP 50 groups compared to the radiation group (p < 0.001) (Table I).

### Effects of OMP on sperm DNA integrity 

The results of independent *t* test showed that the percentage of sperm with fragmented DNA significantly increased in the radiation group compared to the control and R+OMP 30 groups (p < 0.001, p < 0.05). In addition, all OMP groups significantly decreased the percentage of DNA-fragmented sperm in comparison with the radiation group (p < 0.001) (Table I).

### Serum testosterone level

**Figure 2:**
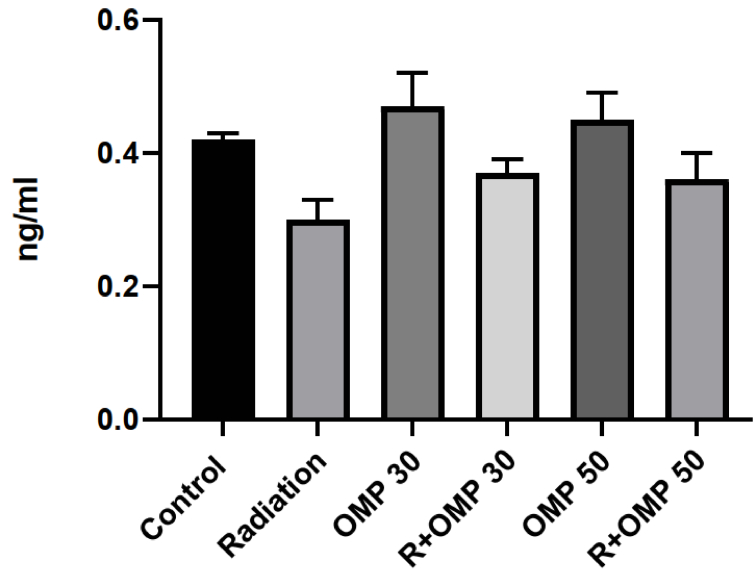
Serum testosterone level, R: Radiation, OMP: Omeprazole.

Despite these obvious differences in the serum testosterone levels between the control and radiation groups at 1 wk after irradiation (on day 15 of the study), the serum testosterone levels were not significantly different among the groups (Figure 2).

### Histopathological findings

Considering the histopathological analysis light microscopic examination of the testicular tissue sections, results in the control group showed normal seminiferous tubules with the complete process of the spermatogenic series in stepwise stages of development (type A and B spermatogonia, primary and secondary spermatocyte, spermatid, and spermatozoa) and Sertoli cells.

The multiple seminiferous tubules were separated by intervening connective tissue containing blood vessels, Leydig cells, and other components of connective tissue. There was a normal height of the germinal epithelium with normal cellular associations located in invaginations or dilations between Sertoli cells. In contrast to the control group, results showed that ionizing radiation causes negative effect on the architecture of testes leading to a potent side effect on qualitative and quantitative changes.

**Figure 3:**
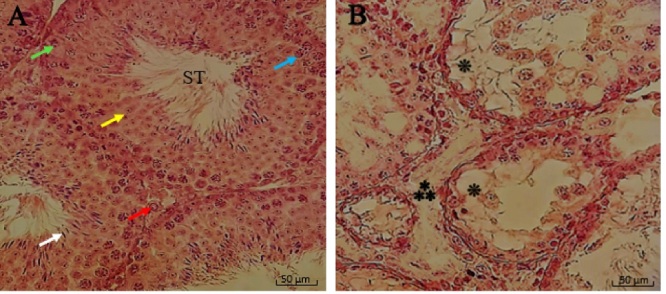
Photomicrograph testis sections in the A) Control group and the B) Ionizing radiation group. Normal testicular structure and spermatogenesis are seen in the control mice group. In contrast, nearly all lineage of spermatogenic cells were arrested, and the spermatozoa were completely depleted of the seminiferous tubules (ST). They observed several vacuolations and widespread edematous areas in the ionizing radiation group. Seminiferous tubules, spermatogonia (blue arrow), spermatocytes (yellow arrow), Spermatid (white arrow), Sertoli cell (green arrow), Leydig cells (red arrow), edematous areas (asterism mark 0.8ex*** ), vacuolation (flower punctuation mark * ) (Magnification: × 400, H&E staining, Scale bar: 50 µm).

Testicular tissue slides of the radiation group exhibited widespread edematous areas accompanying severe testicular atrophy with spermatogenic arrest in the developmental stage and abnormal cytoarchitecture vacuolation. Further, the germinal epithelium was thinner and showed a decrease in height in the radiated mice. The seminiferous tubules had an irregular size and distorted shape with depleted spermatogonia, spermatocytes, and reduction in spermatozoa density. Also, there were some detached, dense irregular nuclei, retained elongated spermatids, and displacement of Sertoli cell (Figure 3).

**Figure 4:**
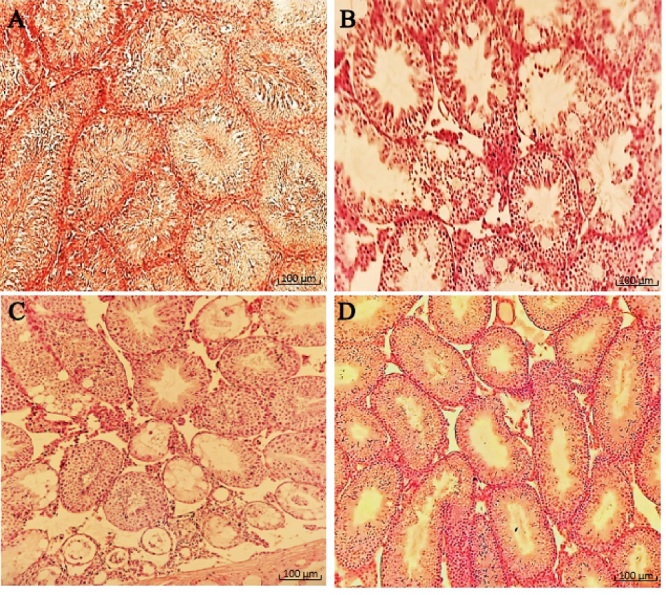
Radioprotective effect of OMP on mice spermatogenesis system induced by ionizing radiation. A) Control, B) Radiation, C) OMP 30 mg treatment, D) OMP 50 mg treatment, control group showed normal seminiferous tubules with the complete process of the spermatogenic series. Meanwhile, irradiation led to severe testicular atrophy with spermatogenic arrest and abnormal cytoarchitecture vacuolation and interstitial edema, while OMP treatment reversed relatively radiation toxicity, especially in OMP 50 mg treatment group (magnification: × 100, H&E staining, scale bar: 100 µm).

Connective tissue containing dilated and congested vessels and Leydig cells were decreased between the seminiferous tubules of ionizing radiation mice. As compared to the ionizing radiation group, examination of the radiated testis with OMP treatment groups (30 and 50 mg dosages) revealed the presence of a decrease in disorganization in the seminiferous tubules with resumption of different stages of the spermatogenic series and relatively showed normal cellular associations and counts. Furthermore, Sertoli cells increased in the seminiferous tubules of treated mice, and some of the seminiferous tubules had a normal height of germinal epithelium. Meanwhile, in some areas, there was tubal vacuolation with abnormal cytoarchitecture and reduction in spermatids or completely depleted spermatozoa; this condition was seen more in the 30 mg OMP treatment group compared to the 50 mg treatment group. Although treatment with OMP reversed the testes histological parameters damage compared to the ionizing radiation group, the interstitial spaces among the seminiferous tubules had increased compared to normal testis. They observed scanty connective tissue containing Leydig cells with interstitial edema, especially in the 30 mg OMP treatment group (Figure 4).

**Table 1:** Sperm parameter and DNA integrity in different groups

**Parameters**	**Control**	**Radiation**	**OMP 30**	**OMP 50**	**R+OMP 30**	**R+OMP 50**	**P-value**
**Count (10^6^)**	57.8 ± 25.4 ${\;}^{\#}$	29.60 ± 14.06 ${\;}^{*}$	49.40 ± 8.2	52 ± 10.7	38.17 ± 12.6	51 ± 8.7	0.04
**Progressive motility (%)**	47.33 ± 3.5 ${\;}^{\#\#\#}$	35 ± 3.74 ${\;}^{***}$	44.4 ± 2.4 ${\;}^{\#\#}$	41.8 ± 1.6 ${\;}^{*\#\#}$	40.6 ± 1.9 ${\;}^{*\#\#}$	41.1 ± 1.7 ${\;}^{*\#\#}$	< 0.001
**Nonprogressive motility (%)**	9.8 ± 2.04	12 ± 3.1	9.4 ± 1.1	9.2 ± 1.5	8.8 ± 1.4	9.5 ± 1.6	0.17
**Immotile (%)**	42.8 ± 3.9 ${\;}^{\#\#\#}$	53 ± 4 ${\;}^{***}$	46 ± 2.3 ${\;}^{\#\#}$	49 ± 1.8	48 ± 2.7	49 ± 4.1 ${\;}^{*}$	< 0.001
**Morphology (%)**	9.17 ± 1.4 ${\;}^{\#\#}$	4.8 ± 1.3 ${\;}^{*}$	8 ± 2.4	7.8 ± 3.03	7.1 ± 1.9	6.8 ± 0.75	0.02
**Viability (%)**	70.0 ± 4.0 ${\;}^{\#\#\#}$	56.4 ± 5.45 ${\;}^{***}$	66.6 ± 1.14 ${\;}^{\#\#\#}$	67.20 ± 2.28 ${\;}^{\#\#\#}$	62.17 ± 4.02 ${\;}^{*}$	66.5 ± 1.87 ${\;}^{\#\#\#}$	< 0.001
**DNA fragmentation (%)**	17.3 ± 2.16 ${\;}^{\#\#\#}$	28.4 ± 3.9 ${\;}^{***}$	21.0 ± 0.7 ${\;}^{\#\#\#}$	19.8 ± 0.8 ${\;}^{\#\#\#}$	22.17 ± 1.16 ${\;}^{*\#\#\#}$	20.33 ± 1.04 ${\;}^{\#\#\#}$	< 0.001
Data are presented as Mean ± SD, One-way ANOVA and Tukey's post-tests, ${\;}^{*}$ P < 0.01, ${\;}^{***}$ P < 0.001 significant differences vs. control group. ${\;}^{\#}$ P < 0.05, ${\;}^{\#\#}$ P < 0.01, ${\;}^{\#\#\#}$ P < 0.001 significant differences vs. radiation group. OMP: Omeprazole, R+OMP: Radiation+Omeprazole							

## 4. Discussion

The main findings of this study were: I) radiation reduced sperm count, progressive motility, and the percent of sperm with normal morphology; II) radiation increased the number of immotile sperm and DNA fragmentation; III) OMP has a radioprotective effect on sperm's progressive motility; viability and DNA fragmentation; IV) radiation caused no significant changes in serum testosterone level, V) OMP showed a protective effect on histopathology of irradiated testis tissue.

Despite the widespread use of radiation for diagnosis and treatment in various medical domains, it can cause adverse effects on normal tissues. Among these, the testis represents one of the most radiosensitive tissues in animals (22). In contrast with the increasing incidence of cancer survivors, the importance of testis protection is highly valued, particularly in those of reproductive age. Several studies have shown the acute and late effects of radiation on sperm parameters. In the present study, the mean total sperm count reduced from 115.9–30.0 ( × 10^6^) after radiation exposure. The progressive motility also decreased considerably from 31.6–11.8%, which was in accordance with another study in this area. Moreover, there was a remarkable decrease in the average frequency of sperm with normal morphology and motile sperm in the present study, which was consistent with the result of a study (23).

Sperm DNA fragmentation following pelvic irradiation is widely studied. It is well known that sperm chromatin differs structurally and functionally from somatic cell chromatin: it lacks nucleosome organization. Sperm DNA is significantly more compacted, with 6-fold higher density and 40-fold less volume compared to somatic cell DNA. Despite the compact packaging and antioxidant protection from seminal fluid, DNA damage can still occur in both developing and mature sperm (24). The findings of an article show 365% more DNA damage after 4 Gy irradiation than the control group (25). Also, our results showed just over an 11% rise in DNA damage in the radiation group compared to the control.

In a study of 2 Gy irradiation, the authors reported a decrease in spermatogenic cells and thinning in the germinal epithelium wall resulting from exposure to ionizing radiation (26). In agreement with a previous study, our findings revealed a spermatogenic arrest in the developmental stage and thinning of germinal epithelium in addition to a decrease in the height of some seminiferous tubules in the radiation group. Additionally, severe testicular atrophy and widespread edematous were observed. Moreover, disorganization and abnormal cytoarchitecture vacuolation were seen. It is widely recognized that radiation has a late adverse effect on serum testosterone levels and can decrease serum testosterone levels significantly during months or years after the end of radiation therapy (27–29). However, there are rare studies that investigate the serum testosterone levels in an acute phase after radiation therapy. In a study, no significant decrease was observed in serum testosterone levels after stereotactic body radiation therapy in prostate cancer (30).

Additionally, another study reported no changes in testosterone levels after proton therapy was statistically significant at the treatment completion point. In the present study, we found no significant change in serum testosterone levels between the control and radiation groups on day 15 of the study. Both dosages of OMP did not have a statistically significant effect on serum testosterone levels. One of the limitations of this study is the relatively small sample size of serum specimens, which may not be sufficient to support the results of statistically significant differences in testosterone tests.

PPIs, commonly used to treat acid-related disorders, have been studied for their potential radioprotective properties. The radioprotective mechanism of PPIs involves several pathways that can reduce radiation-induced damage to healthy tissues: reduction of oxidative stress (ionizing radiation generates reactive oxygen species [ROS]) that cause oxidative damage to cellular components, including DNA, lipids, and proteins. PPIs like OMP, lansoprazole, and pantoprazole have been found to exhibit antioxidant properties (30, 31) and anti-inflammatory effects. PPIs have been shown to inhibit pro-inflammatory cytokines (such as tumor necrosis factor-alpha and interleukin-6) (32). The apoptosis inhibition of PPIs can protect healthy cells by inhibiting radiation-induced apoptosis. A study suggest that PPIs may enhance DNA repair mechanisms, which is vital in minimizing radiation-induced genomic damage. Therefore, OMP as a PPI, due to its antioxidant and anti-inflammatory properties, is a suitable choice for radioprotective studies (14).

To the best of our knowledge, no study evaluates the effectiveness of OMP against testicular damage. In this study, both dosages of 30 and 50 mg/kg of OMP significantly increased the sperm's progressive motility parameter compared with the radiation group (p < 0.01). In addition, there was a considerable rise in sperm viability at both doses of OMP in comparison with the radiation group (p < 0.05). Moreover, DNA fragmentations were significantly lower in G4 (R+OMP 30 mg/kg) than in the radiation group (p < 0.001).

Radiation can cause several histopathological changes in testicular tissue, spermatogenic cell damage and reduction in spermatogenesis due to damage to spermatogonia and spermatocytes, testicular atrophy, and tubular damage to seminiferous tubules, including fluid accumulation and several vacuolations in the interstitial spaces (6).

All of this evidence can be seen in the present study. The histopathological results showed that ionizing radiation caused a significant alteration in the testicular structure parameters, as evident nearly all lineage of spermatogenic cells were arrested, and the spermatozoa were completely depleted of the seminiferous tubules and were observed in several vacuolation and widespread edematous areas. Morphometric examination of the testes showed that OMP could preserve seminiferous tubules of testes in mice that received irradiation. In this study, data showed that the most protective effect of OMP is at a dose of 50 mg/kg in the testes exposed to irradiation.

Radiation generates ROS, leading to oxidative stress and cellular damage (6). OMP has been shown to scavenge free radicals, reduce oxidative stress and enhance the activity of endogenous antioxidant enzymes such as superoxide dismutase and catalase, which neutralize ROS. OMP can modulate the release of these cytokines, thereby reducing inflammation and inhibiting the nuclear factor kappa B signaling pathway, which plays a key role in inflammation and cell survival after radiation exposure (32). Testicular tissue from OMP-treated, irradiated animals shows less structural damage and improved spermatogenesis compared to untreated, irradiated group.

OMP exhibits potential as a radioprotective agent for testicular tissue by mitigating oxidative stress, reducing inflammation, and enhancing cellular survival.

## 5. Conclusion

The findings of this study showed that OMP has a radioprotective effect on sperms progressive motility and viability. In addition, administration of OMP can reduce sperms DNA fragmentation. Furthermore, this study marks the first writing of the radioprotective effects of OMP against testicular damage. This study's findings are promising to be used in clinical practices as radioprotective agent in radiotherapy. Nevertheless, considering the findings of this study, further investigations are warranted, and additional human studies are necessary to elucidate the protective role of OMP in mitigating radiation-induced acute testicular toxicity.

##  Data Availability

Data supporting the findings of this study are available upon reasonable request from the corresponding author.

##  Author Contributions

J. Biabani-Ardakani, S.J. Hosseinimehr, A. Nabi, A. Hassanpour Dehnavi, and H. Nikukar contributed to the conception and design of the work and interpretation of data. J. Biabani-Ardakani, F. Mazaheri, A. Hassanpour Dehnavi, M. Shabani, and F. Sadeghian-Nodoushan carried out the drug preparation, irradiation, specimen collections, and histopathological analysis. M. Hemati carried out the serum testosterone test. A. Nabi carried out the sperm parameters evaluations. Drafting of the manuscript: J. Biabani-Ardakani, A. Nabi, A. Hassanpour Dehnavi, and N. Momeni. All authors read and approved of the final manuscript.

##  Conflict of Interest

The authors declare that there is no conflict of interest.
